# Correction: Complexing agent study *via* computational chemistry for environmentally friendly silver electrodeposition and the application of a silver deposit

**DOI:** 10.1039/d1ra90079j

**Published:** 2021-02-11

**Authors:** Anmin Liu, Xuefeng Ren, Bo Wang, Jie Zhang, Peixia Yang, Jinqiu Zhang, Maozhong An

**Affiliations:** State Key Laboratory of Urban Water Resource and Environment, School of Chemical Engineering and Technology, Harbin Institute of Technology Harbin 150001 China mzan@hit.edu.cn +86-451-86418616 +86-451-86418616; Faculty of Engineering, Architecture and Information Technology, School of Chemical Engineering, The University of Queensland St Lucia Brisbane QLD 4072 Australia

## Abstract

Correction for ‘Complexing agent study *via* computational chemistry for environmentally friendly silver electrodeposition and the application of a silver deposit’ by Anmin Liu *et al.*, *RSC Adv.*, 2014, **4**, 40930–40940, DOI: 10.1039/C4RA05869K.

The authors apologize for Fig. 7 being incorrectly shown in the original manuscript. The corrected figure is shown as follows.

**Fig. 7:**
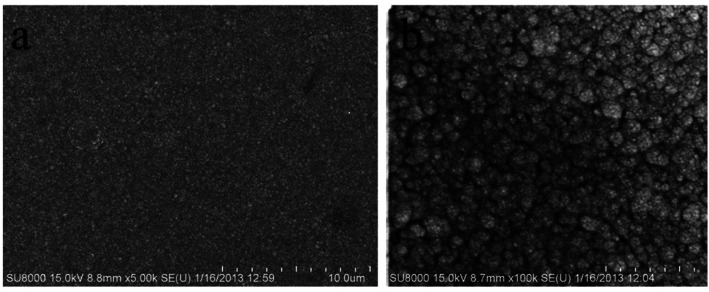
SEM images of the top views of silver deposits obtained from the silver electroplating bath introduced in this work, (a) 5000 times magnification (b) 100 000 times magnification.

The Royal Society of Chemistry apologises for these errors and any consequent inconvenience to authors and readers.

## Supplementary Material

